# Case of the Diffuse Form of Xanthogranulomatous Pyelonephritis

**DOI:** 10.1155/2013/936035

**Published:** 2013-01-09

**Authors:** Tateki Yoshino, Hiroyuki Moriyama

**Affiliations:** Department of Urology, JA Onomichi General Hospital, 1-10-23 Hirahara, Hiroshima, Onomichi 722-0018, Japan

## Abstract

Xanthogranulomatous pyelonephritis (XGP) is a rare entity and constitutes less than 1% of chronic pyelonephritis. A 71-year-old male was introduced to our department with general malaise and abnormal findings of computed tomography (CT). Abnormal findings of complete blood count and laboratory examination included an elevated WBC count and C-reactive protein. Urinalysis showed combined hematuria and pyuria, and *Escherichia coli* was detected in urine culture. Abdominal CT revealed left hydronephrosis with staghorn renal calculi and thin cortex of the left kidney. Because of poor condition, the patient underwent construction of the left nephrostomy. After that, the remission of the inflammation was achieved. Several months after the construction, frequent obstructions of nephrostomy catheter because of turbid urine and intermittent fever elevation were observed. The patient and his family desired left nephrectomy despite his poor condition in general. Surgical dissection was very difficult due to fixed mass. Not long after that the patient died due to sepsis and cardiovascular failure. Microscopic findings of the left kidney revealed infiltration of lymphocytes and lipid-laden macrophages (xanthoma cells) corresponding to XGP.

## 1. Introduction

Xanthogranulomatous pyelonephritis (XGP) is a variant of chronic pyelonephritis which is frequently associated with urinary tract obstruction and nephrolithiasis. XGP is characterized by diffuse or focal, chronic, and severe renal parenchymal infection leading to destruction and replacement of the renal parenchyma by lipid-laden macrophages (xanthoma cells) which impart yellowish tan to the tissue [1]. The confirmatory diagnosis of this entity is based on histopathological examination, and nephrectomy remains the treatment in almost all the cases. We report an unusual case of XGP with cervical spine injury.

## 2. Case Report

A 71-year-old male was introduced to our department with general malaise and abnormal findings of computed tomography (CT). His activity of daily life is keeping to his bed because of cervical spine injury. He had underwent cystostomy and exchange of the catheter at regular intervals. In addition, his past medical history was an operation for the gastric cancer with lung metastasis.

The results of physical examination were unremarkable. Abnormal findings of complete blood count and laboratory examination included an elevated WBC count (9,110/*μ*L) and C-reactive protein (15.1 mg/dL). On the other hand, fasting blood sugar and hemoglobin A1c were within normal limits. Urinalysis showed combined hematuria and pyuria, and *Escherichia coli* was detected in urine culture.

**Figure 1:**
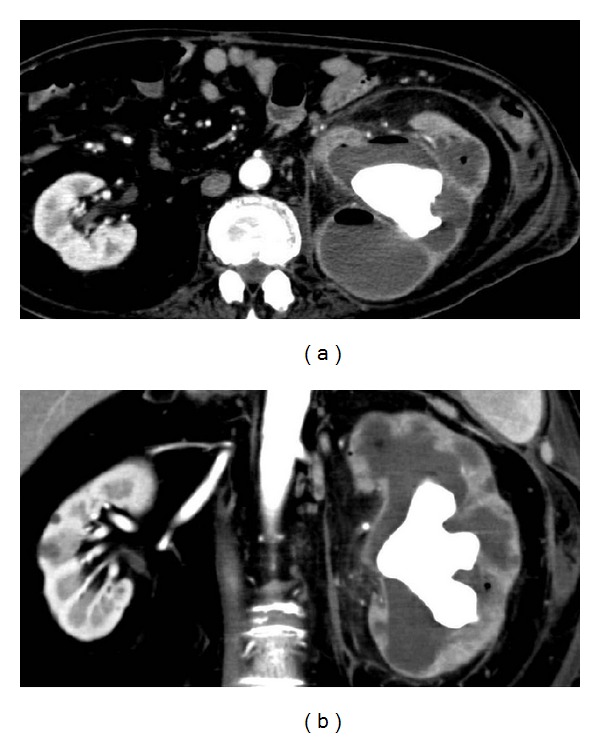
CT findings of the abdomen ((a) transverse section and (b) coronal section) CT showed an enlarged nonfunctioning left kidney and staghorn calculi, with distention of the collecting system.

Abdominal CT revealed left hydronephrosis with staghorn renal calculi and thin cortex of the left kidney (Figure 1). Because of poor condition, the patient underwent construction of the left nephrostomy. After that, remission of the inflammation was achieved, and nephrostomy catheter was also exchanged with cystostomy.

**Figure 2:**
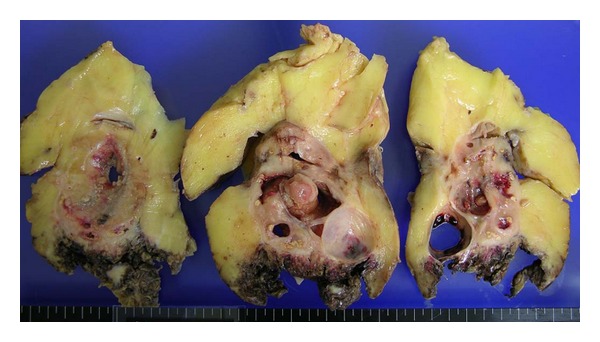
The transverse section of the left kidney after formalin fixation revealed the dilated collecting system and thin cortex, which were surrounded by yellowish fatty tissue.

**Figure 3:**
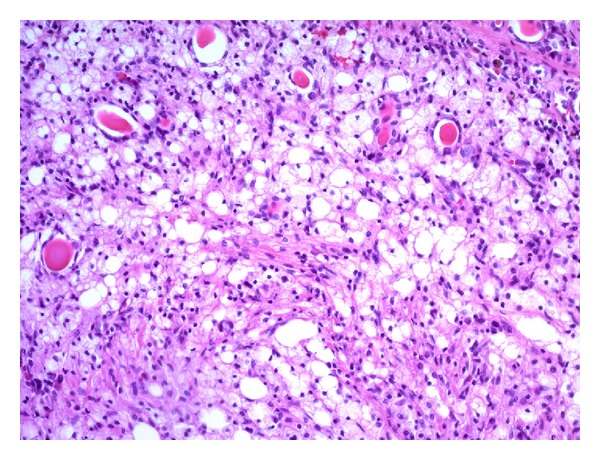
Microscopic findings of the left kidney revealed infiltration of lymphocytes and lipid-laden macrophages (xanthoma cells).

Several months after the construction, frequent obstructions of nephrostomy catheter because of turbid urine and intermittent fever elevation were observed. The patient and his family desired for left nephrectomy despite of poor condition in general. After obtaining informed consent, left nephrectomy by open surgery was performed. Surgical dissection was very difficult due to fixed mass. Because of uncontrolled bleeding due to frequent, extensive adhesions, the patient received massive transfusions. Not long after that the patient died due to sepsis and cardiovascular failure. The transverse section of the left kidney revealed the dilated collecting system and thin cortex, which were surrounded by yellowish fatty tissue (Figure 2). Microscopic findings of the left kidney revealed infiltration of lymphocytes and lipid-laden macrophages (xanthoma cells) corresponding to XGP (Figure 3).

## 3. Discussion

XGP is a rare entity and constitutes less than 1% of chronic pyelonephritis. XGP is severe and chronic renal inflammatory condition, generally associated with urinary tract infection and obstructing renal calculi, leading to diffuse or focal kidney destruction [2]. It starts within the pelvis and calyces and subsequently spreads into renal parenchyma; if uncontrolled, it spreads to adjacent tissue and destroy it.

The symptoms are various and include fever, flank pain, and weight loss. Urinary symptoms can include dysuria, frequency, and hematuria.

The complete blood count and laboratory examination will usually confirm leukocytosis and might reveal elevated C-reactive protein, erythrocyte sedimentation rate, or liver enzyme.

XGP is a chronic inflammatory condition of the kidney that is increasingly well recognized. The disease process is characterized by the destruction and replacement of renal parenchyma by lipid-laden macrophages (xanthoma cells). Schlangenhaufer firstly described the disease in 1916 [3]. Only a few series of cases have been published [4–6]. Though the exact pathogenesis of XGP is still not known, but a number of predisposing factors have been implicated. In the previous reports, these included recurrent urinary tract infection, genitourinary obstruction, and nephrolithiasis as the main factors responsible for the development of XGP. Additionally, urolithiasis has been observed in 70% of patients with XGP, and half of them had staghorn calculi. Previous study has also reported XGP patients with diabetes mellitus, transplanted kidney, receiving hemodialysis for chronic renal failure [7, 8]. Positive urine culture was present in about 70% of patients. Although *Escherichia coli* and *Proteus species* are mostly isolated organisms in the urine, *Staphylococcus* and *Pseudomonas* can also be isolated more rarely [9]. The lesion is generally unilateral with both the right and the left kidneys affected with equal frequency. Bilateral lesions, although rare, have a fatal outcome [10]. The present case was unilateral, and the affected kidney was nonfunctional.

The disease process affects the whole of the kidney; focal forms are rare. In other words, two forms of XGP, a diffuse form (85%) and a focal (localized, segmental) form (15%), are well identified [11]. The former is often misdiagnosed as a life-threatening abscess, while the latter is sometimes referred to as the tumor-like form of XGP because the clinical findings are easily confused with those of a renal tumor. In the present case, the diffuse form of XGP was observed. XGP is generally a disease limited to the affected kidney, but spread to adjacent tissues has also been seen. According to the extent of involvement of the adjacent tissue, Malek and Elder [12] have classified this disease into three stages. Stage I: nephric, disease is confined to renal parenchyma only. Stage II: nephric and perinephric, disease process involves renal parenchyma along with perinephric fat. Stage III: nephric and perinephric, disease is extending into adjacent structure or diffuse retroperitoneum. In stage III, psoas abscess and the formation of nephrocolonic, nephrocutaneous fistulae have been described.

The radiologic findings included an enlarged kidney with poor or absent function and with simple or staghorn calculi. Some believe that CT is the most useful imaging modality for identifying XGP. In the diffuse form of XGP, CT might show a classic “bear paw sign” from dilated caliceal spaces, which revealed a rim enhancement [13]. The renal parenchyma was replaced by multiple hypodense, egg-shaped areas representing dilated calices and abscess cavities filled with pus and debris. With the high predilection of spread to contiguous tissue, especially to the peri- and paranephric spaces, psoas muscle, spleen, and formation of nephrocolonic and nephrocutaneous fistulae, the role of CT is vital regarding the evaluation of disease extension.

Whereas the first-line treatment of choice for both, localized and diffuse XGPs, is conservative (appropriate antibiotic treatment), the approach to surgical treatment is different: nephron-sparing surgery is warranted in localized XGP, but diffuse XGP will require nephrectomy with resection of all other involved tissue in most cases. Early diagnosis and prompt treatment play a crucial role in minimizing the morbidity and mortality rates of XGP. Unfortunately, the present case died due to sepsis and cardiovascular failure soon after surgery. Fully informed consent including fatal outcome should be obtained before nephrectomy for chronic pyelonephritis, such as XGP.
